# QTL and PACE analyses identify candidate genes for anthracnose resistance in tomato

**DOI:** 10.3389/fpls.2023.1200999

**Published:** 2023-08-04

**Authors:** Carlos Lopez-Ortiz, Umesh K. Reddy, Chong Zhang, Purushothaman Natarajan, Padma Nimmakayala, Vagner Augusto Benedito, Matthew Fabian, John Stommel

**Affiliations:** ^1^ Department of Biology, Gus R. Douglass Institute, West Virginia State University, Institute, WV, United States; ^2^ The Genetic Improvement for Fruits & Vegetables Laboratory, United States Department of Agriculture, Agricultural Research Service, Beltsville, MD, United States; ^3^ Division of Plant & Soil Sciences, West Virginia University, Morgantown, WV, United States

**Keywords:** anthracnose, tomato, QTL mapping, QTLseq, PACE marker

## Abstract

Anthracnose, caused by the fungal pathogen *Colletotrichum* spp., is one of the most significant tomato diseases in the United States and worldwide. No commercial cultivars with anthracnose resistance are available, limiting resistant breeding. Cultivars with genetic resistance would significantly reduce crop losses, reduce the use of fungicides, and lessen the risks associated with chemical application. A recombinant inbred line (RIL) mapping population (N=243) has been made from a cross between the susceptible US28 cultivar and the resistant but semiwild and small-fruited 95L368 to identify quantitative trait loci (QTLs) associated with anthracnose resistance. The RIL population was phenotyped for resistance by inoculating ripe field-harvested tomato fruits with *Colletotrichum coccodes* for two seasons. In this study, we identified twenty QTLs underlying resistance, with a range of phenotypic variance of 4.5 to 17.2% using a skeletal linkage map and a GWAS. In addition, a QTLseq analysis was performed using deep sequencing of extreme bulks that validated QTL positions identified using traditional mapping and resolved candidate genes underlying various QTLs. We further validated AP2-like ethylene-responsive transcription factor, N-alpha-acetyltransferase (NatA), cytochrome P450, amidase family protein, tetratricopeptide repeat, bHLH transcription factor, and disease resistance protein RGA2-like using PCR allelic competitive extension (PACE) genotyping. PACE assays developed in this study will enable high-throughput screening for use in anthracnose resistance breeding in tomato.

## Introduction

Tomato (*Solanum lycopersicum* L.) represents one of the world’s most economically significant vegetable crops, mainly due to its wide range of culinary applications (FAOSTAT, 2019). Moreover, according to the USDA Economic Research Service (ERS), tomato is among the most popular consumed vegetables in the United States, with a total production in 2020 of ~13.5 million tons and crop value totaling an estimated $1.66 billion. In addition, the tomato has a high nutritional value in many diets due to its essential nutrients, including lycopene, beta-carotene, vitamins, and flavonoids (Raiola et al., 2014; Li et al., 2018). Despite its high economic demand and important dietary contributions (Van Bueren et al., 2011), tomato production is constrained by post-harvest losses, including mechanical injuries and fungal infections, that reduce available yield and quality (Arah et al., 2015; Abera et al., 2020). Several *Colletotrichum* species cause anthracnose, *Colletotrichum coccodes* being the most prominent (Stommel, 2001; Liu et al., 2011), and this disease is one of the most common postharvest fruit pathogens (Coates and Johnson, 1997; Singh and Sharma, 2018). It affects more than 470 widespread crop species (Hyde et al., 2009), including yam, lentil, mango, cassava, and soybean (Uddin et al., 2018; Liu et al., 2019; Darkwa et al., 2020; Boufleur et al., 2021; Gela et al., 2021). Among Solanaceous species, anthracnose is considered a severe disease for tomato (Alkan et al., 2015), pepper (Zhao et al., 2020; Mishra et al., 2021), and potato (Liu et al., 2011; Ignatov et al., 2019). A 54–80% global yield loss has been attributed to anthracnose disease, reducing the overall harvest and quality (Garg et al., 2014; Saxena et al., 2016).

*Colletotrichum* can survive as small, black microsclerotia in the soil and as acervuli in plant debris between seasons, thus the difficulty of its eradication (Dillard and Cobb, 1998; Sonavane and Venkataravanappa, 2022). The anthracnose infection mechanism typically begins in immature fruit with the formation of melanized appressoria that penetrates the fruit cuticle. It then forms specialized structures resembling biotrophic-like assemblages (Cannon et al., 2000; Latunde-Dada, 2001). *Colletotrichum* species remain quiescent until the fruit ripens; at this point, the fungus initiates necrotrophic infection, culminating in disease development (Prusky et al., 2013). Many control approaches are available to prevent or limit crop disease and consequential damage caused by fungi. These include using pathogen-free seeds, crop rotation and alternate host weed control, well-drained soil, and repeated fungicide applications (Dowling et al., 2020; Konsue et al., 2020; Ciofini et al., 2022). Fungicide use is prevalent for the control of anthracnose despite several drawbacks, including cost-effectiveness, environmental pollution, pathogen resistance, and residue contamination in the market produce (Guo et al., 2019).

Moreover, the current demand for sustainable tomato production has increased the interest in the nonchemical control of pests and diseases, emphasizing the importance of breeding for improved yield, nutrition, and host resistance or tolerance to biotic and abiotic stresses (Van Bueren et al., 2011). Anthracnose resistance was noted in the small-fruited introduction PI 272636 of wild tomato germplasm, which also showed resistance to six other *Colletotrichum* species, including *Colletotrichum coccodes* (Barksdale, 1972). However, efforts to transfer resistance into market types have been challenging because of the polygenic and complex inheritance observed in the resistant germplasm (Stommel and Haynes, 1998; Stommel, 2001). Nonetheless, little is understood about the QTLs and genomic regions harboring them, limiting the development of marker assays that can facilitate high-throughput screening. Hence, in this study, we resolved the positions of QTL/genes and related polymorphisms essential for developing and implementing marker-assisted breeding of anthracnose resistance.

Utilizing Genotype by Sequencing (GBS) for making linkage maps for the use of QTL mapping and QTLseq using deep sequencing of extreme bulks is very efficient and economical for genetic analysis in several crop species (Michelmore et al., 1991; Deokar et al., 2019). Combining whole-genome resequencing with conventional BSA, QTL-seq efficiently identifies genomic areas linked with the trait of interest (Takagi et al., 2013; Zou et al., 2016). This research aimed to identify genomic regions linked to anthracnose resistance using QTL mapping using a skeletal map with a recombinant inbred line (RIL) population and a subsequent QTL-seq. This study also aimed to develop PACE (PCR allelic competitive extension) assays for the candidate genes underlying various QTLs for high-throughput breeding lines and germplasm screening.

## Materials and methods

### Developing a recombinant inbred line (RIL) mapping population

The development of a recombinant inbred line (RIL) population from the cross of two parent lines, US28 (Rio Grande) and 95L368 (PI 272636), was carried out as described by (Stommel, 2001). Two hundred forty-three RILs were developed and carried to the F_7_ generation from the F_2_ generation through the single-seed descent method. The parent lines and RILs were germinated in a greenhouse and then transplanted to a field at the USDA-ARS Beltsville Agricultural Research Center in Beltsville, Maryland, USA. A field plot technique was adopted using four replications. Fresh leaves were collected from each RIL and both parents, lyophilized, and stored at -80°C until use.

### Inoculum preparation and pathogen inoculation

Mature red fruits from each RIL and parental line were harvested and transferred to shaded benches in a greenhouse. The harvested fruit from each line, with a mean of 22 fruits per block per line, were subjected to *Colletotrichum* infection. The infection was induced by injecting a droplet of *C. coccodes* inoculum (5 × 10^6^ conidia/mL) into the fruit and puncturing through the droplet with a needle to a depth of approximately 2 mm (Barksdale and Koch, 1969; Robbins and Angell, 1970; Stommel and Haynes, 1998). Fruits infected with distilled water were used as control. A daytime temperature of 28 ± 4°C and a night temperature of 18°C were maintained. The diameter of the lesion spread on the fruit was measured in millimeters after six days after inoculation.

### DNA isolation and genotyping

We genotyped 243 RIL samples using the genotyping-by-sequencing (GBS) technology using the protocol established by (Elshire et al., 2011). DNA was isolated from the leaf of each RIL adopting the DNeasy Plant Mini Kit (Qiagen, USA). *ApeK*I restriction enzyme was used to digest genomic DNA, which was then ligated to barcoded adapters. The adapter-ligated libraries of all the samples were pooled and amplified with Illumina sequencing primers. The Bioanalyzer 2100 (Invitrogen, USA) and the Qubit 4 fluorometer (Invitrogen, USA) were employed to evaluate the GBS library’s quality and quantity, respectively. The library was sequenced using paired-end sequencing technology on the Illumina NextSeq500 platform. The bcl2fastq program was used to convert the resulting bcl image files to FASTQ with 2x75 bp reads (Illumina, CA, USA). Raw sequences were first quality-filtered by FASTX‐Toolkit (http://hannonlab.cshl.edu/fastx-toolkit) and trimmed to eliminate low-quality bases with a quality score of less than 20 from the ends of reads. Reads with 30% low-quality scores (Q < 15) were eliminated. The GBS reads were de-multiplexed, and variants were called with a new workflow using the GB-eaSy tool, which has the advantage of utilizing paired-end reads from GBS data to call variants (Wickland et al., 2017). The tomato genome build SL3.0 (https://solgenomics.net/organism/Solanum_ lycopersicum/genome/) was used as a reference for the variant calling. The obtained variants in vcf format were utilized for conventional QTL mapping and GWAS analysis.

### Construction of a skeletal map

Linkage analysis and map construction of SNPs generated by GBS involved use of MultiPoint (http://www.multiqtl.com) (Ronin et al., 2015) based on reduction of the mapping problem to the traveler salesperson and solution heuristic algorithms based on Evolutionary Strategy optimization (Mester et al., 2003; Mester et al., 2004; Korol et al., 2009; Reddy et al., 2014). GBS resulted in a disproportion between the high number of scored markers for the mapping populations and population size. MultiPoint analysis allows for selecting the most informative markers for building a reliable skeletal map, whereas other markers are anchored to a skeletal framework map (Mester et al., 2003). For building a skeletal map, we selected error-free markers based on the presence of “twins” (i.e., markers with zero distance) in the dataset. This approach derives from the expectation that because of genotyping errors, the probability of finding false recombinants between absolutely linked markers is higher than observing absolute linkage for closely (but not absolutely) linked markers. The major steps of the algorithm for building ultra-dense genetic maps implemented in MultiPoint include: a “delegate” marker selected from each twin group (including markers with zero distance); except for the twins of various groups, all remaining markers are moved to a heap; delegate markers are ordered to linkage groups (LGs); possible gaps in the LGs are filled by using markers from the heap that belong to twin groups of lower size or singleton markers; and map stability is tested by jack-knife resampling followed by removal of markers violating local map stability and/or monotony (i.e., deviation from the expected increase of recombination rate between a marker and its subsequent neighbors along the map). The last step attaches the markers from the heap to the skeletal map. Each heap marker is attached to the skeletal map if its distance to the closest interval does not exceed the length of this interval. The genetic linkage map was graphically displayed by use of MapChart2.2 (Voorrips, 2002).

### QTL mapping

Genotypic data were classified as “a” (P1 type, homozygous resistance), “b” (P2 type, homozygous susceptibility), “h” (heterozygous), and “x” (missing) for use in QTL analysis. QTL analysis was carried out using the interval mapping (IM) approach using the MapQTL 6 (https://www.kyazma.nl/index.php/ MapQTL) (Ooijen, 2009) and MultiQTL (https://multiqtl.com) (Korol et al., 1998). In MultiQTL analysis, we adopted “two-linked-QTL model”, as described by (Korol et al., 2009). QTL analysis was performed independently for each season. In addition, a multi-environment analysis (MEA) was also performed by simultaneously examining both seasons. This approach allowed for a comprehensive assessment of QTLs across the environments. A multiple interval mapping (MIM) approach was employed to decrease further the residual variance associated with each QTL while permuting the presence and absence of each QTL located on other chromosomes. This method improved the accuracy of the QTL mapping process and enhanced the precision of QTL detection and validation (Kao et al., 1999). Further, using 10,000 permutation runs, the significance of QTL effects was estimated, and in addition, the standard error of the QTL effects was calculated using an additional 10,000 bootstrap runs.

### Population structure and genomewide association study (GWAS)

We used 61,046 SNPs from GBS data for principal component analysis (PCA) to resolve population structure. Genotype positions in PCA were color-coded based on anthracnose lesion diameter. The eigenvalues were estimated by using the EIGENSTRAT algorithm (Patterson et al., 2006) with SNP & Variation Suite (SVS v8.1.5) (Golden Helix, Inc., Bozeman, MT, USA; www.goldenhelix.com). A multiple-locus mixed linear GWAS model developed by the Efficient Mixed-Model Association Expedited method was used to identify SNPs associated with lesion development. Manhattan plots were made to visualize the associated SNPs using GenomeBrowse v1.0 (Golden Helix, Inc), and a false discovery rate (FDR) using the Benjamini-Hochberg method (Sesia et al., 2021) was employed to analyze the strength of association.

### QTLseq

Resistant(R) and susceptible (S) bulks were made, each with 30 extreme resistant and susceptible individuals, for use in QTL-seq. Equimolar concentrations of genomic DNA from the individual samples were combined to create the resistant and susceptible bulks to build libraries using the NEBNext Ultra II DNA Library Prep Kit for Illumina (NEB) following the manufacturer’s instructions. DNA was fragmented to have an average insert size of 450 bases, and the ends were repaired; subsequently, Illumina adapters were added to the fragmented and repaired DNA. The adapter-ligated DNA libraries were further amplified and sequenced on NextSeq500 system (Illumina, USA) to generate paired-end reads with a read length of 2x150 bp and sequencing coverage of more than 250X to the genome.

The raw sequencing reads from the bulks were quality filtered to remove adapters and low-quality bases using Trimmomatic (Bolger et al., 2014). The quality-filtered FASTQ files (paired-end) for each of the bulks were mapped using Burrows-Wheeler Alignment (BWA) to the tomato reference genome build SL4.0 (https://solgenomics.net/organism/Solanum lycopersicum/genome/), and the whole genome alignment was performed using ‘mem’ with default parameters (Li and Durbin, 2009). The resulting BAM file from the genome alignment was sorted using Picard tools v. 2.18.27 (https://broadinstitute.github.io/picard/) and indexed using SAMtools v. 1.15.1 (http://samtools.sourceforge.net/). Using Picard tools version 2.18.27, duplicates were detected, and read groups were created (https://broadinstitute.github.io/picard/). The GATK tool ‘haplotypecaller’ (https://gatk.broadinstitute.org/) was used with the default settings to call variants from the sorted BAM file. The resultant variant calling file (VCF) was used to generate the SNP index to identify the closely linked SNPs and QTLs for the target trait in each bulk. SNP-index and Delta SNP-index for each bulk were calculated using QTL-seq (https://github.com/YuSugihara/QTL-seq) (Takagi et al., 2013). The corresponding SNP-index graphs were plotted for both the bulks to identify candidate genes. KEGG Orthology-Based Annotation System (KOBAS) (http://kobas.cbi.pku.edu.cn/) was employed for KEGG pathway mapping form genes identified in QTL mapping and QTLseq, while dot plots were created using the ggplot2 package in R (Bu et al., 2021).

### PACE-based SNP genotyping

For PCR allelic competitive extension (PACE), allele-specific primers were made (Integrated DNA Technologies, Inc). Twenty four SNP sites were targeted for genotyping the individual RILs used in two extreme bulks. The PACE PCR components included 5 µL of PACE Genotyping Master Mix (2X) (3CR Bioscience, Essex, UK) containing FAM, HEX, and ROX fluorophores, 0.11 µL primer assay mix (72X), 2 µL template DNA, and 3 µL of molecular biology grade water. The PCR conditions were adopted in three stages as follows: (1) one cycle at 94°C for 15 min, (2) 10 cycles each including template denaturation (94°C, 20 seconds) and annealing/extension with a drop of 0.8°C per cycle (65 to 57°C, 60 s), (3) 30 cycles each comprising denaturation at 94°C for 20 s, and annealing/extension at 57°C, for 60 s (3crBioscience, United Kingdom). Further, 3 to 9 cycles of final denaturation and annealing/extension were performed to increase the amplification and generate dense, well-separated clusters. Genotypes were clustered using StepOne plus auto caller (Applied Biosystems). For developing PACE markers, sequences with determined physical positions were selected based on QTLseq results.

## Results

### Disease response of the mapping population

**Figure 1:**
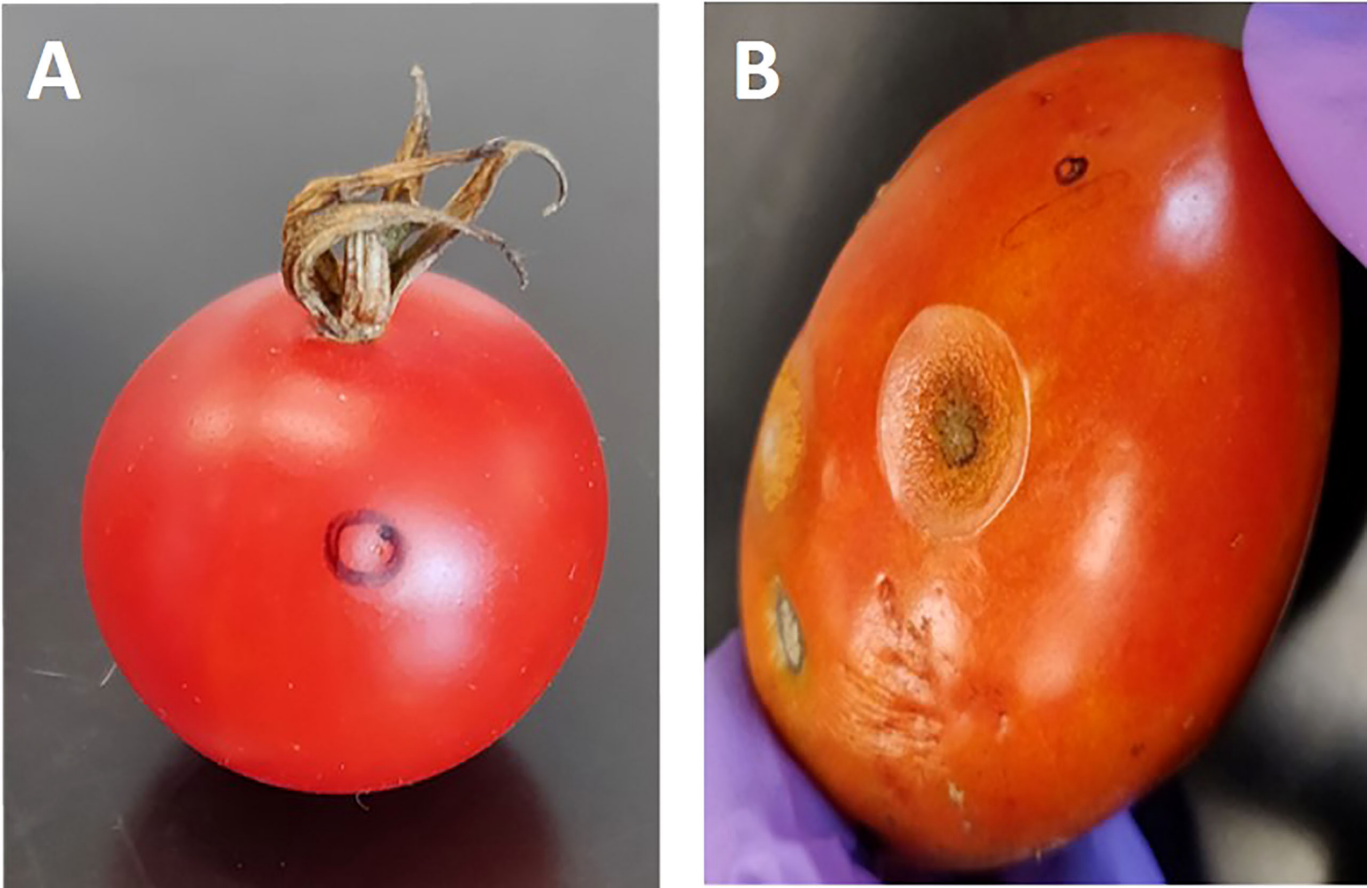
Tomato fruits of two-parent lines of the recombinant inbred lines (RILs) population at the ripening stage after anthracnose infection. Fruit of resistant parent line 95L368 **(A)** showing an absence of lesion development and susceptible parent line US28 **(B)** displaying large lesion at the ripening stage after six days of inoculation with anthracnose (*C. coccodes*) using a hypodermic puncture technique.

The parental lines “US28” (susceptible parent; *Solanum lycopersicum* var. *lycopersicum*) and small-fruited “95L368” (resistant parent), which exhibit characteristics of *Solanum lycopersicum* var. *cerasiforme*, along with the (RIL) population derived from their cross, were subjected to inoculation. The disease severity was evaluated for two seasons based on the lesion diameter on ripened fruits, as shown in **Figure 1**. The analysis of variance (ANOVA) conducted for both seasons revealed that there was no significant difference in disease severity between the seasons (*p*>0.05); therefore, an average of lesion development from both seasons was used for further analysis. The susceptible cultivar “US28” showed lesion sizes in the range of 13.37–18.05 mm, while the resistant cultivar “95L368” showed smaller (0.91-0.99 mm) lesions, exhibiting significant differences in disease severity (Student’s t-test; *p*<0.001) between resistant and susceptible parental lines (**Table S1**). The frequency distribution of lesion diameter across the RILs in both seasons exhibited polygenic distribution and was further confirmed by testing for normality (*p*<0.001) using Shapiro–Wilk test. The frequency distribution of disease severity of the RIL population was skewed towards the resistant parent line 95L368 (**Figure S1**).

### GBS, SNP analysis, and map construction

**Figure 2:**
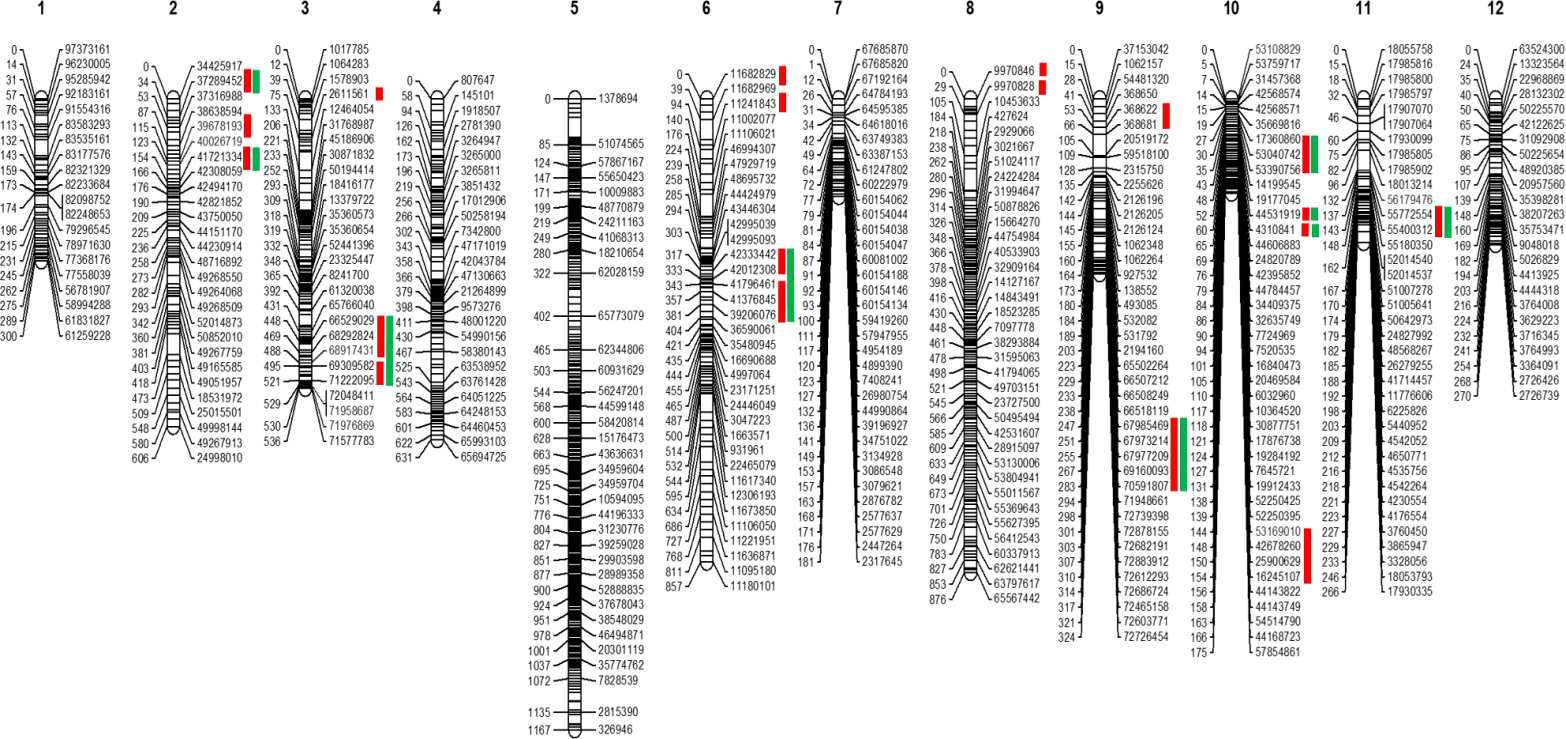
Skeletal map of tomato. The positions of loci are specified on right side of each linkage group. Red bars at the right side indicate the position of the QTLs. In contrast, green bars show these QTLs are common in the QTLseq analysis.

The GBS resulted in 61,046 SNPs, of which 15,300 were polymorphic between parental genotypes (95L368 and US28) (**Table S2**). To select skeletal markers, SNPs violating map stability on mapping were removed and linkage groups were reanalyzed several times until the map showed complete stability. Use of MultiPoint allowed for detection and removal of markers violating the order stability and monotonic growth of distances in the skeletal map. After cleaning, markers from the heap were checked as candidates for filling-in the gaps. The map showed a strong threshold of the absolute linked markers and showed very good correspondence between the map characteristics (the number of skeletal markers and length of the map). Of the 1610 SNPs selected for building the skeletal map by Multipoint analysis, 357 could not be assigned to specific chromosome locations in the *Solanum lycopersicum* genome sequence because of their distorted segregation. Thus, a subset of 1253 high-quality and informative SNP markers was integrated into the 12 tomato chromosomes (**Table S3**) to construct a skeletal genetic map. Chromosomes 1 to 12 contained 58, 78, 117, 120, 368, 102, 35, 175, 40, 55, 37 and 68 skeletal markers, respectively. The total length of the map was 6,189 centimorgans (cM), and the mean marker interval was 4.93 cM between markers. Ten regions on chromosomes 1, 2, 3, 5, 6, 8, 9, and 11, contained wider gaps (more than 30 cM) (**Figure 2**).

### QTL mapping

**Figure 3:**
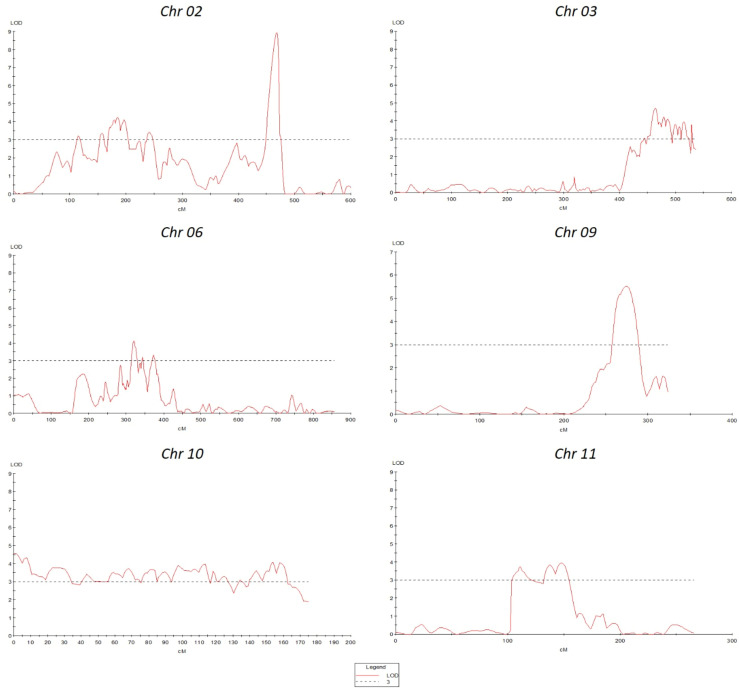
Quantitative trait locus (QTLs) positions on various chromosomes.

QTL analysis was performed using disease severity data (lesion diameter) using both MapQTL and MultiQTL programs. Twenty QTLs were detected on chromosomes 2, 3, 6, 8, 9, 10, and 11 (**Figure 3**). The location of individual QTLs, their significance level, mode of inheritance, and variance explained are in **Table 1**. Similarly, additional information on QTLs detected using the MapQTL and MultiQTL are in **Tables S4**, **S5**, respectively. All the QTLs detected exhibited negative effects, indicating that the causative allele is from the susceptible parent. Four QTLs were on chromosome 2, with phenotypic variances ranging from 4.5 to 8.1%. Highly significant QTL with a LOD score 21.11 was between S3_2463027 and S3_2925032 on chromosome 3 (*SlAnt3.1*), accounting for 17.2% of the phenotypic variance. Furthermore, four QTLs were identified on chromosome 6 (*SlAnt6.1, SlAnt6.2, SlAnt6.3*, and *SlAnt6.4*). The phenotypic variance explained by the four QTLs was 9.1, 5, 6.9, and 6.7%, respectively, and *SlAnt6.2* showed the highest LOD score of 25.91. In addition, QTLs with LOD scores of 8.45 and 4.68 were identified on chromosome 8, explaining 8.2% and 9.5% of the phenotypic variance, respectively. Likewise, on chromosome 9, two QTLs were detected with LOD scores of 5.18 and 10.33, explaining 7.6 to 11.2% phenotypic variance. On chromosome 10, four QTLs were identified with LODs ranging from 3.83 to 9.06, while the percentage of phenotypic variance explained was between 7.5 to 8%. Lastly, one QTL was found on chromosome 11 with 3.95 LOD, explaining 7.3% of the phenotypic variance. Importantly, QTLs were located in several disease resistant genes: alpha/beta-hydrolase superfamily protein; auxin-regulated protein; basic helix-loop-helix (bHLH) transcription factor; cytochrome P450 family protein; disease resistance protein (NBS-LRR class) family; ethylene-responsive transcription factor; F-box protein; kinase family protein; pectin lyase-like superfamily protein and pectinesterase; pentatricopeptide repeat (PPR) superfamily protein; sec14p-like phosphatidylinositol transfer family protein; zinc finger family protein; pathogenesis-related protein 1; and phenylalanine ammonia-lyase; chitinase and chitin-binding protein. Complete details of identified genes within QTL locations are in **Table S6**.

**Table 1:** Quantitative trait loci (QTL) linked with the resistance to anthracnose observed after six days post-inoculation of the 95L368×US28 RIL population.

QTL Name	Chr	Peak Interval (cM)^1^	LOD^2^	Additive^3^	Right flanking marker (Mb)	Center flanking marker (Mb)	PEV^4^
*SlAnt2.1^a^*	2	19.6-35.6	6.23	-1.68	36351550	37289452	4.5
*SlAnt2.2^b^*	2	109.9-115.4	3.34	-1.01	39677456	39678193	6.3
*SlAnt2.3^b^*	2	154.3-159.3	3.3	-1.03	41721334	42370519	6.2
*SlAnt2.4^b^*	2	169.8-196.1	4.25	-1.18	42494400	42854743	8.1
*SlAnt3.1^a^*	3	71.4-91.6	21.11	-1.92	2463027	2762083	17.2
		99.6-105.2		-1.94	2850917	2925032	
*SlAnt3.2^b^*	3	444.3-496.9	4.69	-1.28	66528800	69309620	11.9
*SlAnt3.3^b^*	3	504.2-536.4	4.08	-1.15	69518289	72165672	9.3
*SlAnt6.1^a^*	6	0.8-21	5.42	-0.83	11682829	11682832	9.1
		21.2-50.8		-1.86	11241708	11682832	
*SlAnt6.2^a^*	6	23.6-35.2	25.91	-1.31	11682832	11682969	5
		38.6-64.4		-0.76	11241708	11682969	
*SlAnt6.3^b^*	6	343.3-373.1	3.32	-1.03	39584138	41796461	6.7
*SlAnt6.4^b^*	6	316.8-328.1	3.82	-1.12	42092524	42333442	6.9
*SlAnt8.1^a^*	8	11.5-20.5	8.45	-2.25	9970811	9970835	8.2
*SlAnt8.2^a^*	8	13.6-27.2	4.68	-2.31	9970828	9970835	9.5
*SlAnt9.1^a^*	9	61.2-66.6	10.33	-2.45	368622	368681	7.6
*SlAnt9.2^b^*	9	247-282.7	5.18	-1.36	67973214	70591807	11.2
*SlAnt10.1^b^*	10	60.1-109.6	3.98	-1.13	4310841	7724969	7.5
*SlAnt10.2^b^*	10	0-30.2	4.32	-1.15	12712135	35669816	8
*SlAnt10.3^a^*	10	57.4-60.4	9.06	-2.18	44531919	44606883	7.7
*SlAnt10.4^b^*	10	140.6-160.5	3.83	-1.07	41666022	53759717	7.5
*SlAnt11.1^b^*	11	137.4-147.8	3.95	-1.11	55400312	55772554	7.3

a QTLs identified by MultiQTL.

b QTLs identified by MapQTL6.

^1^Position of the markers on the linkage map in centiMorgans (cM); ^2^LOD, logarithm of odds at the position of the peak; ^3^Additive effect of QTL; ^4^PEV percent of trait variance explained by the QTL.

### Population stratification and genomewide association analysis

**Figure 4:**
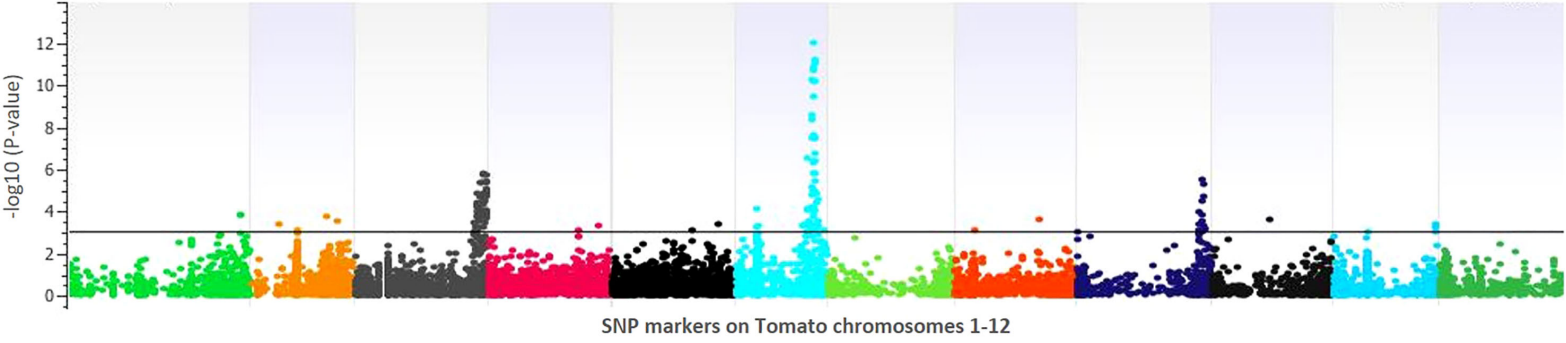
Manhattan plot of the genomewide association study for lesion diameter on tomato. The X-axis displays chromosome coordinates, while the Y-axis shows the negative log-10 of the association P-value for each SNP. A higher negative log-10 value suggests a stronger association with the trait.

Principal component analysis (PCA) was used to examine the distribution of 243 RILs and the parental lines. If the cross in the study is between the genetically close parents, the distribution of RILs expected in PCA would be in two clusters. Since the current study used two genetically distant parents, the distribution of RILs was not confined to the parental clusters but distorted in PCA (**Figure S2**), indicating GWAS might resolve additional associations that did not appear in QTL mapping. Based on this hypothesis, we performed GWAS using a Multi Locus Mixed Model to discover additional SNP associations using the population structure resolved by eigenvectors as a cofactor to minimize spurious associations arising from the confounding effects of population stratification. A variance partition plot can be used to understand the strength of a GWAS model. Variance plots combine proportions explained by total genetic variance, variance explained in the current model, and error. The partition of variance estimated in the present study for lesion diameter was ~60%, indicating the current model’s robustness (**Figure S3**). GenomeBrowse v8.1.5 displayed the Manhattan plots of associated SNPs for lesion diameter (**Figure 4**). **Table S7** shows the GWAS statistics for SNP associations of lesion diameter and their allelic effects, regression beta, and FDR correction.

**Figure 5:**
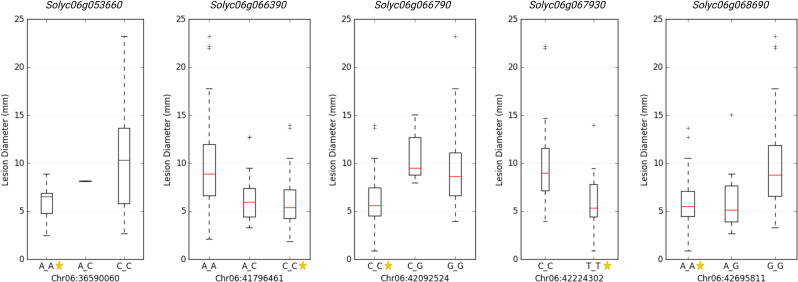
Allelic effects of significantly associated SNPs for anthracnose lesion diameter in tomato. Y-axis represents the lesion diameter in millimeters. * represents the allele from the resistant parent 95L368.

GWAS identified 75 significantly associated SNPs with disease severity across the 12 chromosomes. Chromosome 6 had a major haplotype that consisted of 48 SNPs, with the top SNP in the peak at 41796461^th^ position (p=0.00000; FDR=0.0000), located in the exon of AP2-like ethylene-responsive transcription factor (*Solyc06g066390*). S6_42092524 (p=0.00000; FDR=0.0000), S6_42097760 (p=0.00001; FDR=0.0062) and S6_42097792 (p=0.00001; FDR=0.0048) of the same haplotype belonging to N-alpha-acetyltransferase (*Solyc06g066790*). S6_42279818 (p=0.00000; FDR=0.0010) was in a trafficking protein particle complex (*Solyc06g068030*). Additionally, S6_42224302 (p=0.00000; FDR=0.0000) was in the exon of Cytochrome P450 (*Solyc06g067930*). Another highly associated SNP (S6_42695811; p=0.00000; FDR=0.0000) was in the exon region of Amidase family protein (*Solyc06g068690*). In addition, on chromosome 6, an intergenic SNP (S6_42810399; p=0.00000; FDR=0.0003) lie between *Solyc06g068860* and *Solyc06g068870* genes, encoding an alpha-mannosidase and bHLH transcription factor, respectively. Likewise, S6_36590060 (p=0.00046; FDR=0.0920) and S6_36590061 (p=0.00046; FDR=0.0926) were intronic SNPs of HR-like lesion-inducing protein (*Solyc06g053660*). Further, additional SNPs highly associated with lesion diameter were observed on chromosome 9, of which S9_68120221 (p=0.00031; FDR=0.05) and S9_69160093 (p=0.00001; FDR=0.0026) were exonic SNPs on *Solyc09g076010* (Acyl-CoA N-acyltransferase with RING/FYVE/PHD-type zinc finger protein), and *Solyc09g083060* (RING-type E3 ubiquitin transferase), respectively. Allelic effects of causative SNPs for selected genes associated with anthracnose resistance are in **Figure 5**.

### QTLseq

**Figure 6:**
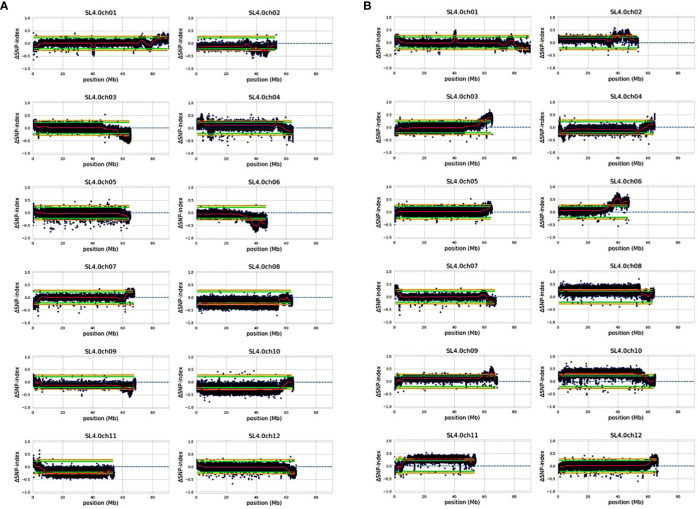
The ΔSNP-index plot was obtained by subtracting the SNP-index from the susceptible to resistant-bulk SNP-index using the reference genome of **(A)** 95L368 resistant parent and **(B)** US28 susceptible parent. The ranges of statistical confidence are indicated in green: P < 0.05 and yellow: P < 0.01.

We constituted resistant and susceptible bulks with equimolar DNAs from 30 RILs with extreme phenotypes manifesting resistance or susceptibility (**Figure S4**). In the RIL population, the lesion diameter ranged from 2.15 (RIL-236) to 20.24 mm (RIL-126) (**Table S1**). We constructed four genomic DNA libraries, specifically two for bulked segregant analysis and two representing the parental lines. These libraries were subjected to Illumina’s paired-end sequencing (2x150 bp) utilizing the NextSeq500 platform. Our sequencing efforts resulted in the generation of 59,214,924 (38X coverage) and 65,172,071 (42X coverage) reads for the parental lines labeled “US-28” and “95L368” respectively. Furthermore, the bulked segregant analysis yielded 1,547,236,596 (296X coverage) and 1,474,242,328 (282X coverage) reads for the susceptible (S) and resistant (R) bulks correspondingly. Through QTL-Seq analysis and alignment to the tomato reference genome (SL4.0), we detected 2,758,048 and 2,774,264 genome-wide SNPs/INDELs for the susceptible (S) and resistant (R) bulks, respectively. A comprehensive summary of the QTLseq results for both the susceptible and resistant bulks is provided in **Supplementary Table S2**. These SNPs and INDELs were subjected to QTLseq analysis to identify causative SNPs by estimating SNP-index. The SNP indices were independently computed for both the resistant and susceptible reference genome assemblies, corresponding to the S-bulk and R-bulk respectively. The average SNP indices of S-bulk and R-bulk and their corresponding ΔSNP-indices were determined with 10 kb increment intervals of 100 kb window. Reference genomes “US-28” and “95L368” were separately used to construct ΔSNP-index plots for all the tomato chromosomes as illustrated in **Figure 6**. Details of all the causative SNPs and corresponding ΔSNPindices, for 95L368 and US-28, are in **Tables S8**, **S9**, respectively.

The genomic regions displaying peaks or valleys in the SNP-index plot or markedly opposing SNP-index trends for both S and R bulks at a cut off 0.5 are considered as QTN associated with the trait (Takagi et al., 2013). Among the 12 chromosomes, chromosome 1 showed fewer SNPs, followed by chromosomes 7 and 12 with 9, and 19 QTNs, respectively. SNP-indices of S-bulk and R-bulk appeared mutually contrasting SNP indices in juxtaposition at the cut-off of 0.5 on chromosomes 3, 6, 9, and 10. For chromosome 3, this genomic region (4.95 Mb) had 183 variants (70 Indels and 113 SNPs) with ΔSNP indices ranging from -0.5 to -0.69. A total of 119 genes were identified on chromosome 3. Similarly, on chromosome 6, a 10.8 Mb (36.39–47.20 Mb) region with a read depth of ≥16 containing 3,179 SNPs showed ΔSNP indices ranging from -0.5 to -0.83. A negative sign of the ΔSNP index indicates introgression from the resistant parent (**Table S8**). Of these 3179 SNPs, 208 SNPs were intergenic, 289 intronic, 92 synonymous, one SNP each in a stop and start codons, 73 in 3’ UTRs, and 50 in 5’ UTRs, 32 indels, 608 downstream, and 1,729 upstream gene variants, while 108 of these were missense variants. This region harbors 607 genes with diverse functional annotations including perception and signaling, membrane transport and trafficking, metabolic processes, and plant stress/defense (**Table S9**). Alpha/beta-Hydrolases superfamily protein, N-alpha-acetyltransferase, Avr9/Cf-9 rapidly elicited protein, bHLH transcription factor, disease resistance protein (TIR-NBS-LRR and RRS1-like class), pathogenic type III effector avirulence factor Avr AvrRpt-cleavage, WRKY transcription factor, cytochrome P450, and AP2-like ethylene-responsive transcription factor are the important disease resistance genes in the list. Additionally, on chromosome 9, two causative genomic regions of 1.39 Mb (58.88-60.28 Mb) and 1.63 Mb (64.03-65.67 Mb) were identified, consisting of 41 SNPs (23 SNPs and 18 indels) containing 13 genes, including auxin-regulated IAA1, F-box protein, kinase interacting (KIP1-like) family protein, and ethylene-responsive factor. On chromosome 10, there were 110 SNPs and 48 Indels with a range of ΔSNP indices from -0.5 to -0.77. Two intergenic SNPs were located between pathogenesis-related protein 1 (*Solyc10g048100*) and unknown protein (*Solyc10g048110*). Likewise, a SNP (S10_43680916) and an Indel (S10_43683659) lie in the intergenic region between the disease resistance protein RGA2-like and a serine carboxypeptidase.

### Colocalized QTLs identified by QTL mapping, QTLseq, and GWAS methods

Of the 20 QTLs identified through traditional QTL mapping, 11 were common with QTLseq and GWAS hits on chromosomes 2, 3, 6, 9, 10, and 11 (**Table 2**). In this study, GWAS analysis revealed significant peaks associated with seven common QTLs. The common QTLs across multiple methods strengthen the evidence for their involvement in anthracnose resistance, highlighting their potential importance. Moreover, among the three methods utilized, a total of 13 genes (*Solyc03g117770, Solyc06g063100, Solyc06g064990, Solyc06g065740, Solyc06g066310, Solyc06g066320, Solyc06g066390, Solyc06g066790, Solyc06g067930, Solyc06g068030, Solyc06g068120, Solyc09g083260*) were common. Additionally, 25 genes were shared between QTL mapping and GWAS, while 35 genes were common between GWAS and QTLseq. **Table S10** presents the complete list of shared genes identified through various methods employed in the study.

**Table 2:** Common QTLs identified for anthracnose resistance in tomato by QTL mapping, QTLseq, and GWAS methods.

QTL Name	Chr	QTL mapping		QTLseq		GWAS	
		Physical interval (bp)		Physical interval (bp)		Physical position (bp)	
*SlAnt2.1*	2	36351550	37289452	34497237	37642033	–	–
*SlAnt2.3*	2	41721334	42370519	41833197	43028031	41025226	–
*SlAnt3.2*	3	66528800	69309620	65000299	65217203	67938049	68936310
*SlAnt6.3*	6	42092524	42333442	42002036	42804465	42092524	42333442
*SlAnt6.4*	6	39584138	41796461	39584072	41567563	39723280	41796461
*SlAnt9.2*	9	67973214	70591807	–	–	67973214	69962504
*SlAnt10.1*	10	4310841	7724969	2727436	8878567	–	–
*SlAnt10.2*	10	12712135	35669816	12218942	35876557	30969068	–
*SlAnt10.3*	10	44531919	44606883	44394795	44959367	–	–
*SlAnt10.4*	10	41666022	53759717	41490715	53011253	–	–
*SlAnt11.1*	11	55400312	55772554	53004886	53401062	55712803	–

Marker physical position based on Heinz1706 tomato reference genome (version of SL4.0).

-, No overlapping QTL positions were shared between analyses.

The identified genes from QTL mapping and QTLseq analyses were subjected to pathway analysis. Genes from QTL mapping showed 19 significantly enriched pathways (p ≤ 0.05) (**Figure S5A**), while in QTLseq genes, 17 pathways were significantly enriched (p ≤ 0.05) (**Figure S5B**). The information regarding these enriched pathways can be found in **Tables S11**, **S12** for QTLmapping and QTLseq genes, respectively. Interestingly, ten common pathways were noted between QTL mapping and QTLseq, including “Basal transcription factors,” “Biosynthesis of secondary metabolites,” “Diterpenoid biosynthesis,” “Glycine, serine and threonine metabolism,” “Metabolic pathways,” “mRNA surveillance pathway,” “Nicotinate and nicotinamide metabolism,” “Oxidative phosphorylation,” “Plant hormone signal transduction,” and “Stilbenoid, diarylheptanoid, and gingerol biosynthesis.” In addition, there were unique pathways detected in QTL mapping, such as “Phagosome,” “Glyoxylate and dicarboxylate metabolism,” “Phenylpropanoid biosynthesis,” “Endocytosis,” and “SNARE interactions in vesicular transport.” On the other hand, QTLseq analysis showed enrichment of “MAPK signaling pathway - plant,” “Monoterpenoid biosynthesis,” “Brassinosteroid biosynthesis,” “Flavonoid biosynthesis,” “Diterpenoid biosynthesis,” and “Nitrogen metabolism.”

### PACE assay genotyping for marker validation

**Figure 7:**
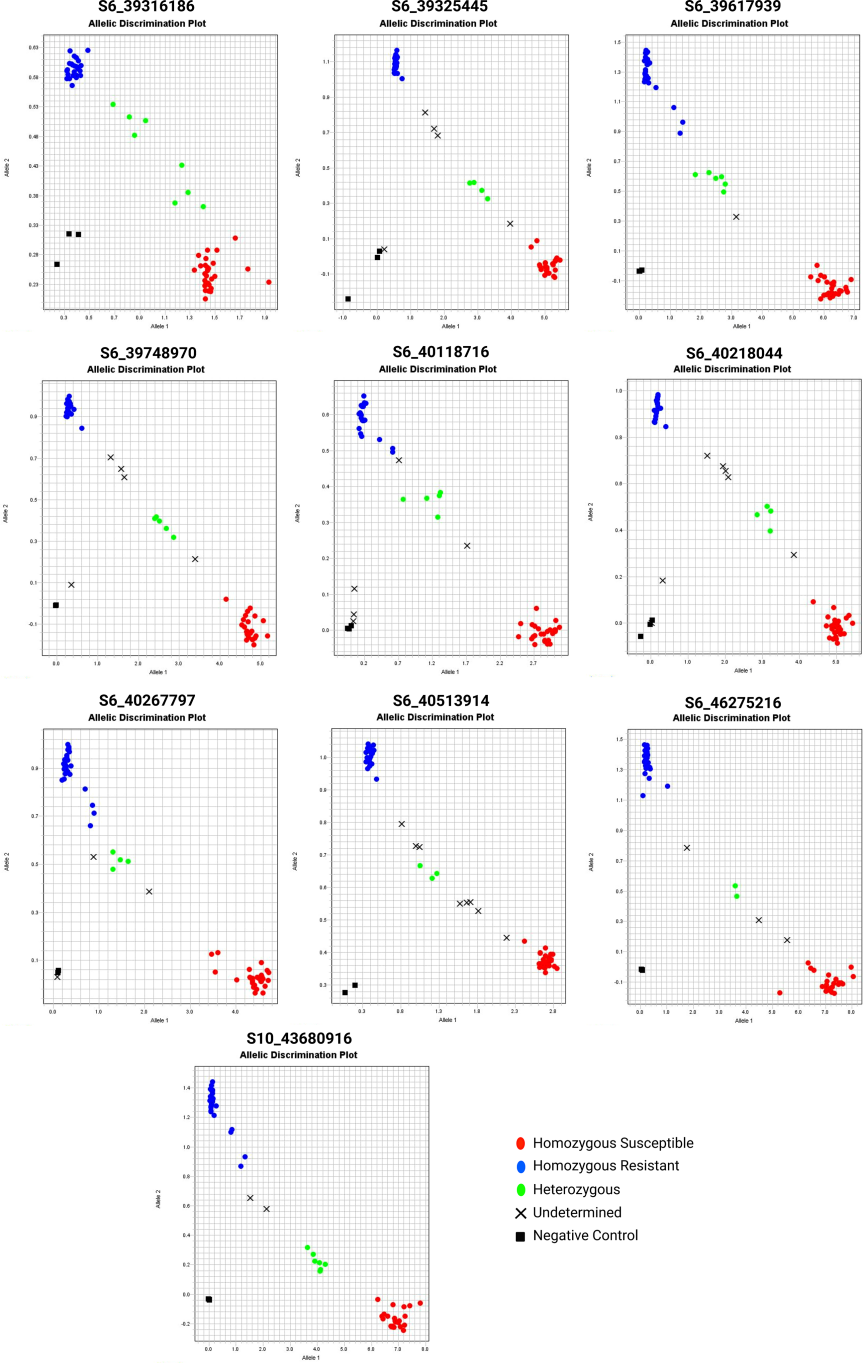
The PACE genotyping assay of selected QTNs from QTLseq. The 60 extreme individuals from QTLseq analysis were evaluated. Each assay also contained three controls without DNA. The red and blue fluorescence intensity was determined in a StepOne Plus system.

We used SNP pairs from QTLseq based on the ΔSNP indices to develop PACE assays consisting of two allele-specific primers (susceptible and resistant parent) and one common reverse primer (**Table S13**). Of 24 PACE markers designed (**Table S14**), only ten assays (S6_39316186, S6_39325445, S6_39617939, S6_39748970, S6_40118716, S6_40218044, S6_40267797, S6_40513914, S6_46275216, and S10_43680916) were informative to resolve three distinct groups - homozygous resistant, heterozygous, and homozygous susceptible (**Figure 7**; **Table S15**). The SNP marker S6_39617939 could resolve resistant and susceptible allelic differences with the highest precision resolving 60 extreme RILs into 28 homozygous resistant, 6 heterozygous, 25 homozygous susceptible alleles, and rest unamplified, respectively. Likewise, S10_43680916 resolved 28 homozygous resistant, 23 homozygous susceptible, and 7 heterozygous RILs. We used non-parametric analyses (Chi-Square, Kruskal-Wallis, and Dunn tests) of genotypes and disease severity for ten PACE assays (**Table 3**). The Kruskal-Wallis test revealed highly significant differences (*P* < 0.01) between A (homozygous resistant), B (homozygous susceptible), and H (heterozygous) groups in all the assays except for S6_39325445. A *post-hoc* analysis using Dunn’s test further confirmed that nine PACE assays in this study are highly effective in discriminating between three groups (A, B, and H).

**Table 3:** Non-parametric tests (Chi-Square, Kruskal-Wallis, and Dunn’s Test) using information obtained from PACE genotyping of tomato RIL population.

Chr	Marker	Kruskal–Wallis Test		Dunn Test (*p*)			Lesion Diameter Mean (mm)		
		*X^2^*	*p*	A-B	A-H	B-H	A	B	H
6	39316186	20.667	<0.001	<0.001	0.3241	0.3874	5.15	12.34	7.71
6	39325445	0.4608	0.7942	0.6701	0.6948	0.5359	7.91	9.53	6.59
6	39617939	24.647	<0.001	<0.001	0.033	0.6895	4.95	12.35	10.52
6	39748970	22.825	<0.001	<0.001	0.0842	0.6067	5.03	12.35	9.96
6	40118716	21.051	<0.001	<0.001	0.2092	0.4122	5.29	12.43	9.73
6	40218044	20.164	<0.001	<0.001	0.378	0.3731	5.06	12.14	8.85
6	40267797	23.898	<0.001	<0.001	0.3172	0.378	4.75	12.14	8.85
6	40513914	21.946	<0.001	<0.001	0.5379	0.3431	4.88	12.14	6.65
6	46275216	14.527	0.0007	0.0005	0.7263	0.8617	6.33	11.39	9.24
10	43680916	13.684	0.0011	0.0032	0.0254	0.6559	6.46	10.88	11.75

P < 0.05 signifies a statistically significant difference between observed and expected, or between groups A, B, and H; A, homozygous resistant; B, homozygous susceptible; H, heterozygous.

## Discussion

*Colletotrichum* spp., a hemibiotrophic fungal pathogen, causes anthracnose, which affects a wide range of crop species, including tomato, during growth and post-harvest, ripening, and storage (Rivera et al., 2016). Traditional approaches for combating anthracnose disease in tomato have proven unsuccessful due to their higher cost and lack of sustainability (Ntui et al., 2021). Although anthracnose resistance was noted in landrace collections, efforts to transfer this trait into elite materials have been unsuccessful due to its complex genetic inheritance (Stommel and Haynes, 1998; Stommel, 2001). Therefore, developing resistance in commercial cultivars using modern genomic approaches, such as genome editing, or by launching genomic selection is the most economical and sustainable strategy. In this study, QTL analysis identified 20 QTLs; we further employed GWAS analysis and QTLseq involving extreme bulks for validation and fine mapping. Results of QTLs identified on chromosomes 2, 3, 6, 10, and 11 in this study, corroborate with previously published other tomato disease and pest studies. *Cf-2* and *Cf-5* conferring resistance to *Cladosporium fulvum* are comparable on genomic position on chromosome 6 (Dixon et al., 1998; Iakovidis et al., 2020). Similarly, the *Mi* locus on chromosome 6 has a common QTL that imparts resistance to three types of root-knot nematodes (Meloidogyne) (Ammiraju et al., 2003). Likewise, QTLs on chromosome 10, responsible for defense against *Phytophthora infestans* are common with the QTLs in the present study (López-Kleine et al., 2013). Thus, colocalized QTLs on chromosomes 6 and 10 may play critical roles in mitigating several diseases and pests.

Plant hormones are pivotal in the dynamic interaction between plants and pathogens (Bari and Jones, 2009). Pathogens can disrupt plant hormone homeostasis during infection or as part of the plant defense response (Svoboda et al., 2021). The plant hormone signal transduction pathways were highly significant in genes identified in our study, indicating similar involvement in response to anthracnose resistance. The important phytohormones that regulate biotic and abiotic stresses are abscisic acid, salicylic acid, jasmonic acid, and ethylene (Nakashima and Yamaguchi-Shinozaki, 2013). In tomato, ethylene plays a crucial role in regulating fruit ripening (Li et al., 2020), which is also involved in the transition of *Colletotrichum* infection from quiescent to necrotrophy (Alkan et al., 2015). Ethylene also promotes the formation of appressorium in *Colletotrichum gloeosporioides* (Ren et al., 2022). Our PACE assay identified homozygous resistant and susceptible alleles of an AP2-like ethylene-responsive transcription factor (*AP2/ERF, Solyc06g066390*). In addition, we also identified nine *ERF* genes on chromosomes 3, 6, 8, and 9 that regulate the ethylene biosynthesis pathway. Overexpression of ethylene-responsive factor 28 (*CaERF28*) caused susceptibility to anthracnose in chile pepper (Mishra et al., 2018). Also, silencing *CaERF28* in pepper fruit through virus-induced gene silencing (VIGS) and CRISPR/Cas9 resulted in a large reduction of transcript levels and substantial repression of fungal load, with no post-infection symptoms of *Colletotrichum truncatum* (Mishra et al., 2019; Mishra et al., 2021). According to these findings, *CaERF28* is a negative regulator of anthracnose resistance in chili pepper. Thus, the *AP2/ERF* identified in our study may also be a candidate for genome editing approach to engineer anthracnose resistance in tomato.

Another process involved in pathogen defense and coupling fungal resistance with changes in the fruit ripening regulatory network is the steroidal glycoalkaloid metabolism (SGAs), which produces tomato defensive compounds such as α-tomatine (Nakayasu et al., 2018; Cárdenas et al., 2019). In tomato, during the transition from green to red fruit, the bitter steroidal glycoalkaloid α-tomatine is transformed into the non-bitter esculeoside A by glycoalkaloid metabolic enzymes (GAME) (Kazachkova et al., 2021; Sonawane et al., 2022). In a recent study, Fabian et al. (Fabian et al., 2022) reported that the resistant 95L368 cultivar shows a higher accumulation of α-tomatine in comparison to the anthracnose-susceptible cultivar (US-28), and resistance was enhanced by targeting *GAME* genes *(GAME5* and *GAME31)*. *GAME31* encodes a 2-oxoglutarate (2OG) and Fe(II)-dependent oxygenase, while *GAME5* encodes a UDP-glycosyltransferase (Nakayasu et al., 2018; Szymański et al., 2020). In the current QTLseq analysis, we located a 2OG oxygenase gene on chromosome 10 (*Solyc10g018190*) and five of them on chromosome 6 (*Solyc06g066830*, *Solyc06g066840*, *Solyc06g066860*, *Solyc06g068250*, and *Solyc06g068270*), all of which showed a negative ΔSNP-index indicating introgression of these genes from the resistant parent 95L368. Likewise, two other significantly enriched pathways in the current study, namely diterpenoid biosynthesis and biosynthesis of secondary metabolites, highlight the synthesis of α-tomatine, a compound known in the defense response against *Colletotrichum* infection. Similarly, Mehmood et al. (Mehmood et al., 2021) reported upregulation of terpenoids and terpene genes in wild strawberry upon challenge with *C. gloeosporioides*.

Cell wall melanization is another process during tomato fruit ripening that helps *Colletotrichum* infection (Páez-Redondo et al., 2022; Silva et al., 2023). *Colletotrichum* appressoria consist of strongly polarized cells from which a needle-like penetration hypha emerges to pierce the cuticle and epidermal cell wall (Howard and Valent, 1996; Latunde-Dada, 2001). The phenylpropanoid pathway controls the lignin synthesis, and over 15 cytochrome P450-dependent (CYP) reactions have been characterized in this pathway (Werck-Reichhart, 1995). Our study identified 13 cytochrome P450 genes (CYPs) on chromosomes 2, 6, 8, and 10. Among these, a specific SNP in the *Solyc06g067930* gene exhibited a strong association and was colocalized across all three methods. Exogenous brassinosteroid enhances plant defense against *C. gloeosporioides* by activating phenylpropanoid pathway in *Camellia sinensis L* (Zhang et al., 2018a). Bared et al. (Barad et al., 2017) reported that the phenylpropanoid pathway was upregulated in RNAi–SlPH tomato line with reduced fruit acidity and higher pH upon *Colletotrichum gloeosporioides* colonization, leading to host susceptibility. Notably, tomato *Sly-CYP86* mutants with reduced cuticle phenotype were highly susceptible to *C. coccodes* fruit infection (Shi et al., 2013). Our study revealed significant enrichment of the brassinosteroid biosynthesis and phenylpropanoid biosynthesis pathways, suggesting that genes involved in these pathways may be crucial in conferring anthracnose resistance in tomato. In similar lines, during colonization of the host, the fungus secretes ammonia, which modifies the pH and influences gene expression in the host, so contributing to pathogenicity (Prusky et al., 2001). The capacity of postharvest pathogens to modify pH locally was first documented for *Colletotrichum gloeosporioides*, and later expanded to include *Alternaria alternata* and *Fusarium oxysporum* (Eshel et al., 2002; Prusky et al., 2004; Miyara et al., 2010; Miyara et al., 2012). It has been reported that auxin (IAA) is often involved in plant-pathogen interaction by regulating plant disease susceptibility and balancing immune responses and plant fitness (Robert-Seilaniantz et al., 2011; Denancé et al., 2013; Chanclud and Morel, 2016; Barbez et al., 2017). In our QTLseq and GWAS analysis, we identified an amidase gene (*Solyc06g068690*) strongly linked with resistance and further validated by PACE. Amidases are indole-3-acetamide amidohydrolase enzymes that catalyze the production of indole-3-acetic acid (IAA) from indole-3-acetamide (Neu et al., 2007). Nida et al. (Nida et al., 2021) reported that the expression of an amidase gene (*Sobic.006G002400*) was upregulated in sorghum grains upon fungus infection. Thus, the susceptible *Solyc06g068690* allele may negatively impact disease resistance to anthracnose in tomato.

Further, the oxidative phosphorylation pathway was significantly enriched in QTL mapping and QTLseq analysis. In plants, oxidative phosphorylation and other diverse biosynthetic pathways typically generate reactive oxygen species (ROS) (Almeida et al., 2015). The rapid accumulation of ROS triggers an oxidative burst, which results in cell death and acts as a defense mechanism to limit the spread of pathogen infection (Apel and Hirt, 2004). Moreover, SNARE interactions in vesicular transport and MAPK signaling pathways were found enriched in QTL mapping and QTLseq, respectively. SNARE proteins are essential components of vesicle fusion in eukaryotes (Kwon et al., 2020); several SNAREs have been shown to be crucial in protecting against powdery mildew and other pathogens in wheat (Wang et al., 2023). Likewise, protein kinases play a pivotal role in mediating protein phosphorylation during pattern-triggered immunity, associated with the perception and signal transduction processes activating immune responses and defense mechanisms against *Colletotrichum* pathogens (Jiang et al., 2022).

In our study, we also discovered several known resistance genes that are likely to contribute to anthracnose resistance in tomato besides the ROS-mediated defense responses (Zhang et al., 2018b). For instance, a basic helix–loop–helix (bHLH) transcription factor (*Solyc06g083170*) that segregated the RIL population into two clusters in the PACE analysis was identified. bHLH TFs have been linked to biotic and abiotic stress responses (Ng et al., 2018). Zhu et al. (Zhu et al., 2022) reported that bHLH13, bHLH25, and bHLH35 genes were rapidly upregulated to *C. truncatum* infection in soybean (*Glycine max*). Similarly, a bHLH TF *GmPIB1* facilitated resistance to *Phytophthora sojae* infection (Cheng et al., 2018). Also, the disease resistance gene RGA2-like (*Solyc10g049230*) was observed in our QTLseq and QTL mapping. We further validated it using PACE. *RGA1* and *RGA2* proteins have been associated with anthracnose resistance loci in beans as part of two clusters of resistance (R) genes previously described (López et al., 2003). We also validated two genes on chromosome 6, *Solyc06g066790* and *Solyc06g069080* encoding a N-alpha-acetyltransferase (NatA) and tetratricopeptide repeat protein (TPR), respectively. Pentatricopeptide repeat proteins (PPR/TPR) were observed in anthracnose resistant sorghum, and were repressed in the susceptible line in response to anthracnose infection (Fu et al., 2020). N-terminal acetyltransferase (Nat) complexes NatA and NatB regulate plant stress responses and mediate drought tolerance and disease resistance in Arabidopsis by co-translationally acetylating 60% of the proteome (Huber et al., 2021).

In conclusion, the loci and putative genes identified in this work will enable genetic improvement of tomato and other crops for anthracnose resistance. However, further study is necessary to fine-map the loci and confirm the potential genes by genome editing. Further study will include gene expression investigations comparing the anthracnose-resistant 95L368 tomato line to the susceptible US28 tomato line to ensure possible gene functions. The efficacy of the PACE markers presented in this work will be of significant value for marker-assisted selection (MAS) in tomato breeding for anthracnose resistance. In addition, further functional research utilizing CRISPR/Cas9 gene knockouts or overexpression approaches will be necessary to establish the role of these potential genes and the underlying mechanism regulating anthracnose resistance in tomato.

## Data availability statement

The datasets presented in this study can be found in online repositories. The names of the repository/repositories and accession number(s) can be found in the article/**Supplementary Material**.

## Author contributions

UR, PNi, VB, and JS were responsible for the conception and design of the experiment. UR, CL-O, PNa, CZ, and MF participated in the material preparation, data collection, and analysis. CL-O and UR drafted the manuscript, and all authors commented on previous versions. All authors have read and approved the final version of the manuscript.
