# Supplementary material for: QTL and PACE analyses identify candidate genes for anthracnose resistance in tomato
Source: Front Plant Sci. 2023 Aug 4;14:1200999. doi: 10.3389/fpls.2023.1200999 (PMC10443646; doi:10.3389/fpls.2023.1200999)
Supplement: Supplementary file 2 [file DataSheet_1.docx]

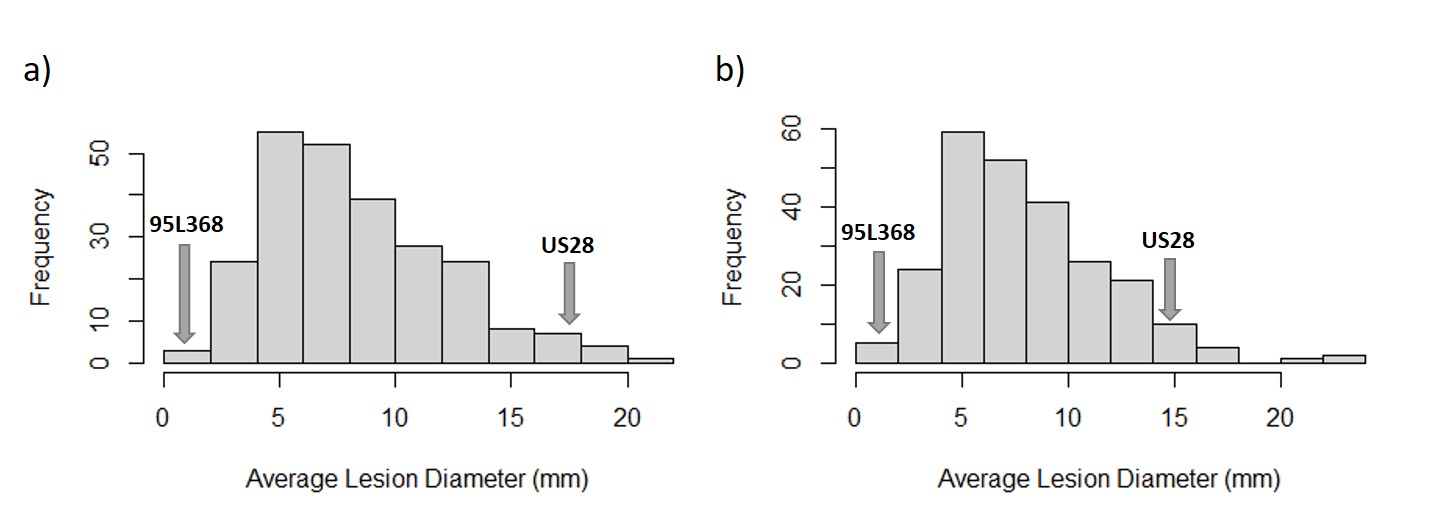


**Figure S1.** Frequency distribution for disease lesion diameter at six days post-inoculation with *C. coccodes* for season 1 (a), season 2 (b) in the 95L368 × US28 RIL tomato population (N = 243).

**
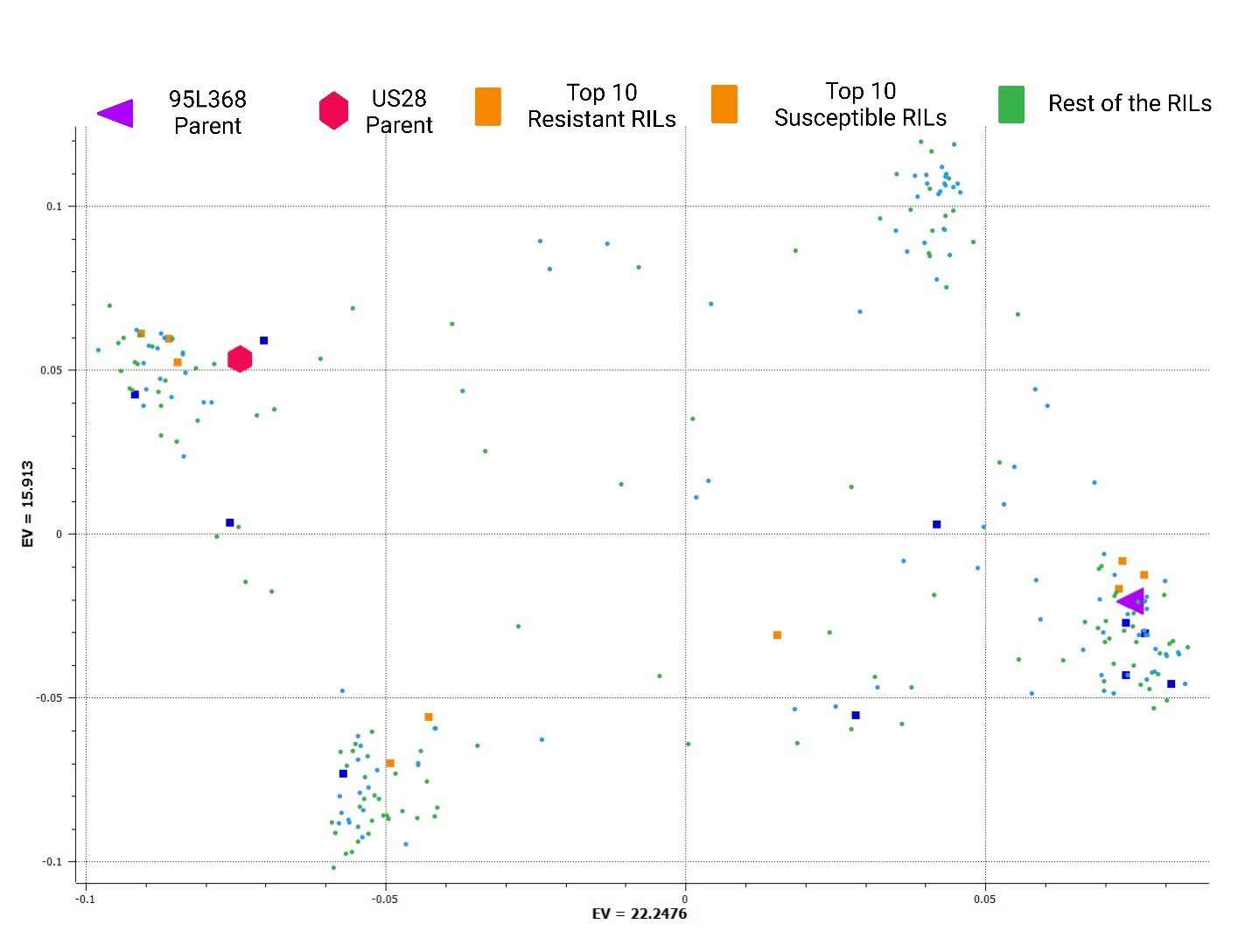
**

**Figure S2.** Principal component analysis showing the genetic distribution of susceptible and resistant parents and 243 RILs according to 61,046 single nucleotide polymorphisms (SNPs).

**
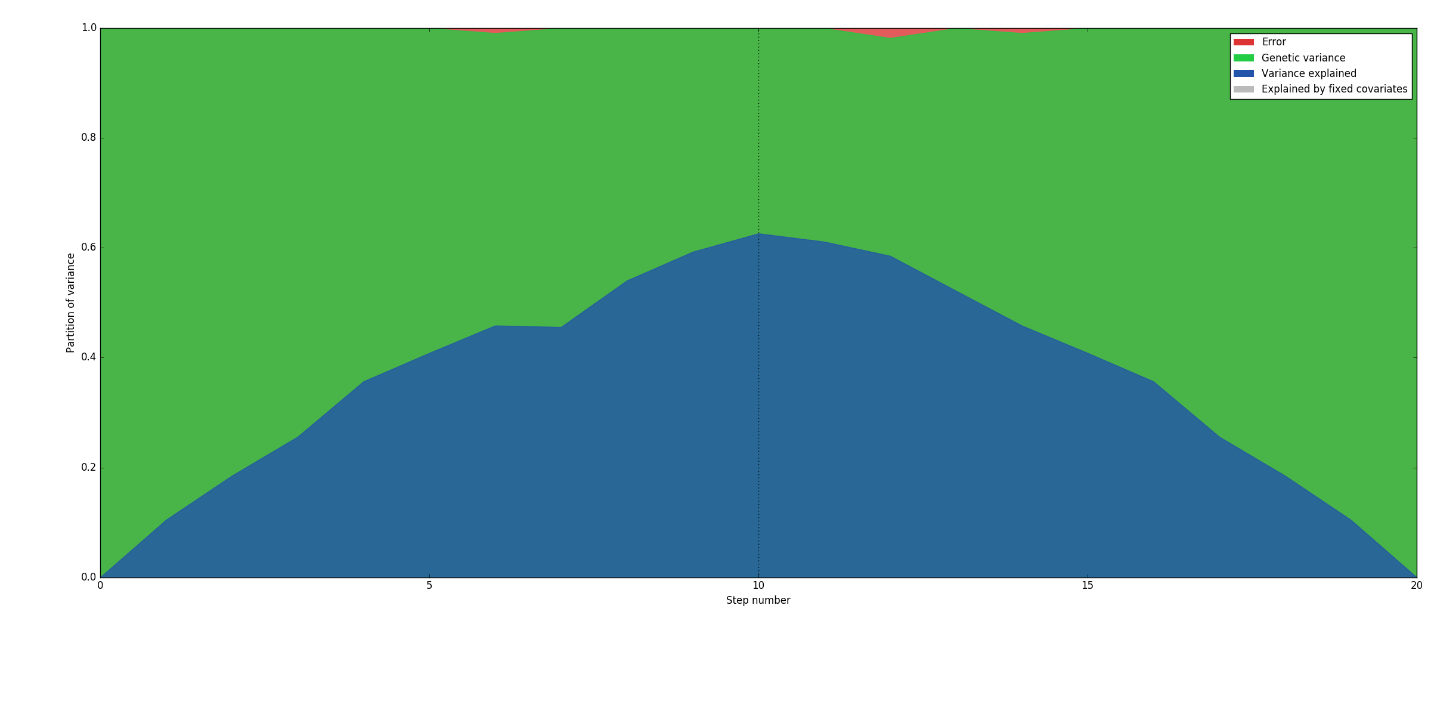
**

**Figure S3.** Variance Partitioning Analysis shows the partitioned error, genetic variance, variance explained in the current GWAS model, and variance explained by covariates.


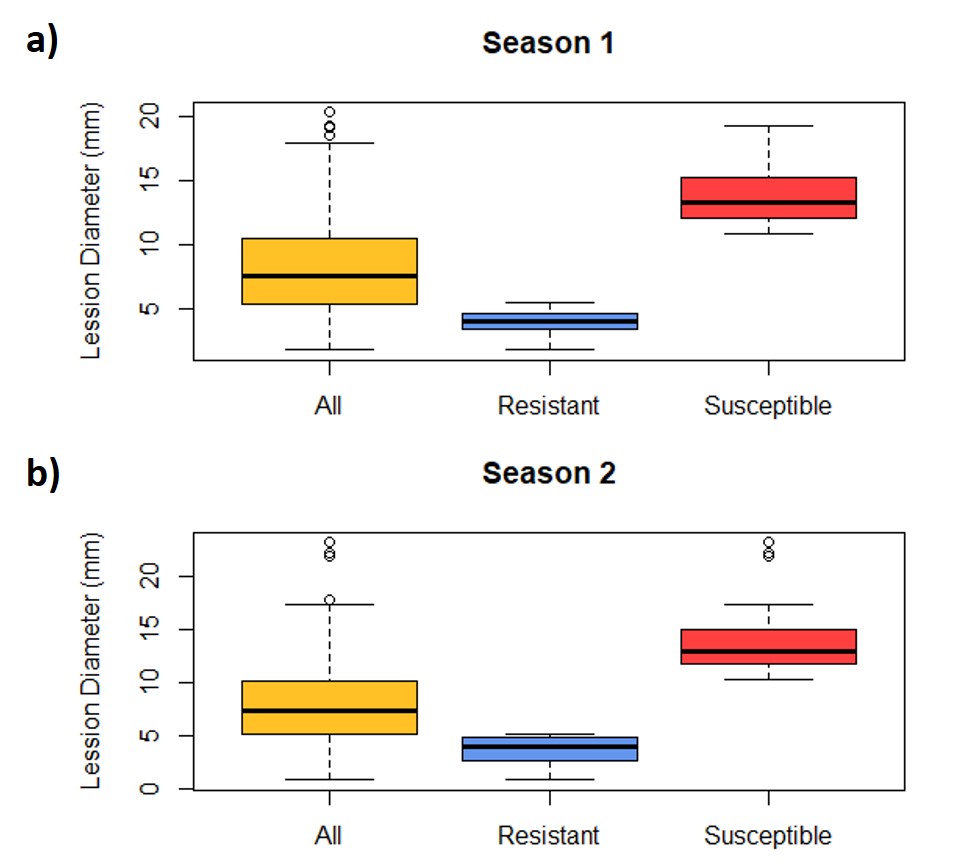


**Figure S4.** Frequency distribution for anthracnose resistance showing phenotypic variation in RIL population. The DNA of 30 RILs with extreme phenotypes (high and low disease scores) in both seasons were used to develop susceptible and resistant bulks sequenced through the QTL-Seq approach.


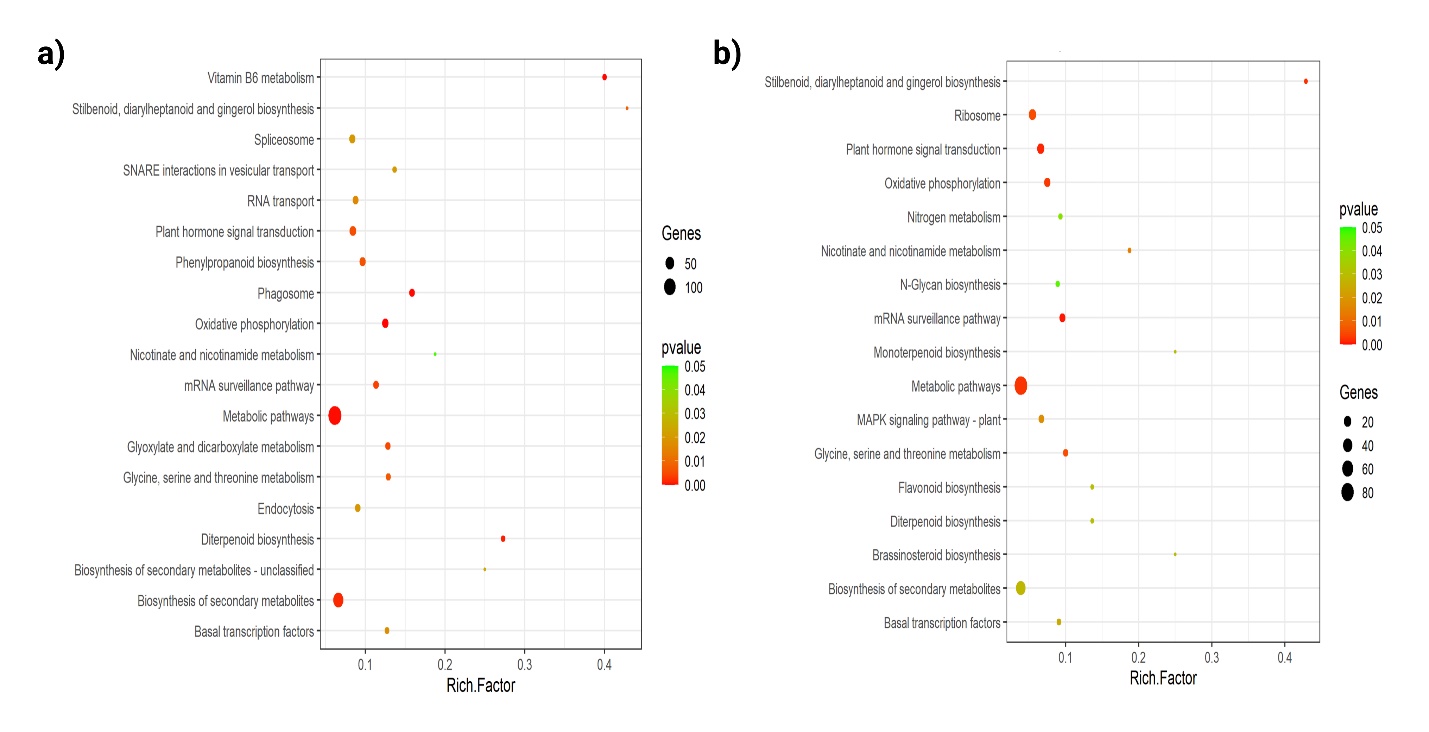


**Figure S5.** Scatter plot of top 20 enriched KEGG pathways among genes identified by QTL mapping (a) and QTLseq (b). The rich factor is the ratio of the number of genes identified to the total gene number in a pathway. The Q-value is a corrected p-value. The color and size of the dots represent the range of q-values and the number of genes mapped to the indicated pathways, respectively. A full list of pathways for QTL mapping and QTLseq can be found in Table S11 and Table S12, respectively.
